# Crystal Structure and Active Site Engineering of a Halophilic γ-Carbonic Anhydrase

**DOI:** 10.3389/fmicb.2020.00742

**Published:** 2020-04-28

**Authors:** Malvina Vogler, Ram Karan, Dominik Renn, Alexandra Vancea, Marie-Theres Vielberg, Stefan W. Grötzinger, Priya DasSarma, Shiladitya DasSarma, Jörg Eppinger, Michael Groll, Magnus Rueping

**Affiliations:** ^1^KAUST Catalysis Center, King Abdullah University of Science and Technology, Thuwal, Saudi Arabia; ^2^Center for Integrated Protein Science Munich, Department of Chemistry, Technische Universität München, Garching, Germany; ^3^Department of Microbiology and Immunology, Institute of Marine and Environmental Technology, University of Maryland School of Medicine, Baltimore, MD, United States

**Keywords:** extremophiles, halophiles, thermophiles, extremozyme, salt adaptation, mutagenesis, gamma-carbonic anhydrase

## Abstract

Environments previously thought to be uninhabitable offer a tremendous wealth of unexplored microorganisms and enzymes. In this paper, we present the discovery and characterization of a novel γ-carbonic anhydrase (γ-CA) from the polyextreme Red Sea brine pool Discovery Deep (2141 m depth, 44.8°C, 26.2% salt) by single-cell genome sequencing. The extensive analysis of the selected gene helps demonstrate the potential of this culture-independent method. The enzyme was expressed in the bioengineered haloarchaeon *Halobacterium* sp. NRC-1 and characterized by X-ray crystallography and mutagenesis. The 2.6 Å crystal structure of the protein shows a trimeric arrangement. Within the γ-CA, several possible structural determinants responsible for the enzyme’s salt stability could be highlighted. Moreover, the amino acid composition on the protein surface and the intra- and intermolecular interactions within the protein differ significantly from those of its close homologs. To gain further insights into the catalytic residues of the γ-CA enzyme, we created a library of variants around the active site residues and successfully improved the enzyme activity by 17-fold. As several γ-CAs have been reported without measurable activity, this provides further clues as to critical residues. Our study reveals insights into the halophilic γ-CA activity and its unique adaptations. The study of the polyextremophilic carbonic anhydrase provides a basis for outlining insights into strategies for salt adaptation, yielding enzymes with industrially valuable properties, and the underlying mechanisms of protein evolution.

## Introduction

Recent years have seen the discoveries of extremophiles in environments previously considered uninhabitable (Madigan and Marrs, 1997; Cavicchioli et al., 2011; Antunes et al., 2017; Jorquera et al., 2019; Merino et al., 2019). To date, enzymes from extremophiles gained increasing attention because they have adapted their structure and retained their function under harsh conditions, where their mesophilic homologs are non-functional (Persidis, 1998; Akal et al., 2019). In particular, these proteins are attractive for biotechnological and chemical industries keen on replacing traditional catalysts with enzymes. As a result, these extremozymes provide a high stereoselectivity with fewer side reactions, and lower the burden on the environment, concomitantly accelerating reaction rates (Littlechild, 2017). However, many processes require high temperatures, use of salts, organic solvents, or other demanding conditions that are incompatible with the stability and function of most proteins (Littlechild, 2015; Amoozegar et al., 2019). Conversely, extremophilic organisms, which are naturally adapted to withstand harsh conditions, provide a perspective for optimization and rational protein-engineering approaches (Liszka et al., 2012).

The Red Sea constitutes a unique habitat of several anoxic deep-sea brine pools (Gurvich, 2006; Behzad et al., 2016) and, therefore, promises the discovery of a vast number of new extremophiles and enzymes. The anoxic environment is polyextremophilic, being filled with water, a high salt concentration, metal content, and elevated temperatures. Additionally, their increased density prevents mixing with the overlying seawater (Gurvich, 2006; Antunes et al., 2011). For example, the Discovery Deep brine pool below 2038 m is characterized by a salinity close to saturation (26.2%, w/v) with a temperature of 44.7°C (Hunt et al., 1967). Therefore, Discovery Deep’s microorganisms have only been scarcely investigated; nonetheless, a recent surge in interest has begun to provide glimpses of the wealth of new information waiting to be explored (Antunes et al., 2011; Mwirichia et al., 2016; Grotzinger et al., 2018).

The limited accessibility and uncultivability of the aforementioned microorganisms with current laboratory techniques hamper the investigation of these exceptional species (Stewart, 2012). Consequently, methods independent of cultivation and their further development, such as the use of Single Amplified Genomes (SAG), are required (Kvist et al., 2007; Rashid and Stingl, 2015). Here, DNA from a single cell is amplified using the Multiple Displacement Amplification (MDA) techniques (Dean et al., 2001) to generate sufficient DNA for sequencing and to avoid the need for cultivation of the respected organisms. To improve the assembly and annotation accuracy for SAG-derived samples, our group developed the Profile and Pattern Matching (PPM) algorithm method (Grotzinger et al., 2014). In this work, we use data from SAG analysis to investigate an extremophilic γ-carbonic anhydrase (γ-CA) from the Red Sea Discovery Deep brine pool. CAs (E.C. 4.2.1.1) are ubiquitous metalloenzymes that catalyze the reversible hydration of carbon dioxide to bicarbonate (CO_2_ + H_2_O ⇋ HCO_3_^–^ + H^+^) (Supuran, 2008). To date, seven classes have been described: α-, β-, γ-, δ-, ζ-, η-, and θ-CAs. These hydrolases differ significantly in both sequence and structure, whilst all catalyzing the same reaction (Ferry, 2013; Del Prete et al., 2016; Supuran and Capasso, 2017). The α-CA class was the first to be isolated and described (Meldrum and Roughton, 1933), whereas the γ-carbonic anhydrase from the thermophilic archaeon *Methanosarcina thermophila* (Cam) was discovered in 1994 (Alber and Ferry, 1994). It was categorized as a new class showing a left-handed parallel β-helix fold, and central metal coordination in the active site by three histidine residues (Kisker et al., 1996). Subsequently, further γ-CAs have been investigated; however, half of them did not show measurable activity (Park et al., 2012; Herrou and Crosson, 2013), and the underlying molecular mechanisms are still controversial.

Here, we report on the first crystal structure of a halophilic γ-class CA (CA_D). The gene was derived from SAG analysis of an uncultured archaeon from the Red Sea Discovery Deep brine pool (Alam et al., 2013; Mwirichia et al., 2016) and was identified using the PPM algorithm (Grotzinger et al., 2014). The gene was expressed in the bioengineered haloarchaeon *Halobacterium* sp. NRC-1. We demonstrate that CA_D indeed encodes a γ-carbonic anhydrase. Activity analysis of structure-driven designed CA_D variants provided insights into the residues constituting the catalytic site.

## Materials and Methods

### Chemicals and Reagents

Restriction enzymes, T4 DNA ligase, and DNA polymerase were purchased from New England Biolabs (Beverly, MA, United States). Chemicals were purchased from Sigma (St. Louis, MO, United States). Water was desalted and purified using a Milli-Q^®^ Academic system (Merck, Darmstadt, Germany).

### SAG Sampling Sites, Sample Preparation, and Genome Annotation

Samples were collected from the Discovery Deep brine pool in the Red Sea (21° 16.98′/38° 03.18′). Cells were sorted using fluorescence-activated cell sorting (FACS), lysed, the whole genome amplified and sequenced (Mwirichia et al., 2016). Genes were annotated using the INDIGO data warehouse system in combination with the profile pattern matching algorithm (PPMA) (Alam et al., 2013; Grotzinger et al., 2014).

### Strains, Plasmids, Media, and Culture Conditions

*Escherichia coli* One Shot TOP10^®^ chemical competent cells were purchased from Invitrogen (Carlsbad, United States). *E. coli* was grown at 37°C in Luria-Bertani (LB) medium supplemented with 100 μg/ml ampicillin. *Halobacterium* strains were cultured in CM^+^ medium containing 4.3 M NaCl and trace metals at 42°C with shaking as previously described (DasSarma et al., 1995). For solid media, 2% (w/v) agar was added. Stock cultures were maintained in glycerol at -80°C. For short-term use, purified cultures were maintained on stock plates at 4°C.

### Construction of the *Halobacterium* Carbonic Anhydrase Knockout Strain

To eliminate background carbonic anhydrase production, *icf*A was knocked out via the *ura*3-based gene deletion method for *Halobacterium* sp. NRC-1. Approximately 500 bp regions flanking the carbonic anhydrase gene (*icf*A) were amplified by crossover PCR (using primers shown in Supplementary Table S5). The resulting amplified crossover PCR fragment was cloned into the suicide vector, pBB400 using flanking *Hin*dIII and *Eco*RI sites incorporated in the primers (Supplementary Table S5) (Berquist et al., 2006). The resulting plasmid, pBB400Δ*icf*A was transformed into *Halobacterium* sp. NRC-1Δ*ura*3 using the standard PEG-EDTA method (DasSarma et al., 1995). pBB400Δ*icf*A transformants were selected by plating on CM^+^ uracil dropout media (HURA), colonies picked and grown in liquid HURA media, and integrant candidates were plated onto 5-FOA-CM^+^ media plates. Knockout candidates were identified by DNA extraction and PCR using flanking primers listed in Supplementary Table S5 (DasSarma et al., 1995; Berquist et al., 2006).

### Construction of the Expression Plasmids

Synthetic genes were codon-optimized using the java codon adaptation online tool JCat (Grote et al., 2005) for *Halobacterium* sp. (strain NRC-1/ATCC 700922/JCM 11081). The optimized genes were ordered from GeneArt (Regensburg, Germany) and cloned into pRK42, which harbors an N-terminal His_6_-tag, *csp*D2 promoter, origins of replication for *E. coli* and *Halobacterium*, and genes for ampicillin and mevinolin resistance for selection in *E. coli* and *Halobacterium*, respectively.

### Expression of the Carbonic Anhydrase Genes in *Halobacterium* sp. NRC-1Δ*ura*3Δ*icf*A and Purification of the Encoded Proteins

Carbonic anhydrase genes containing vectors were transformed into the *Halobacterium* sp. NRC-1 Δ*ura*3Δ*icf*A strain using the PEG/EDTA method (DasSarma et al., 1995; Karan et al., 2013) and transformants were selected by plating on CM^+^ agar plates using mevinolin resistance. For protein production, cells were grown to late log phase (OD_600__*nm*_ of 0.9–1.0) at 42°C in CM^+^ medium supplemented with 20 μg/ml mevinolin. To induce carbonic anhydrase expression, the cultures were further incubated at 15°C for 72 h.

Cells were harvested by centrifugation (6,000 × *g*, 4°C, 10 min) in a 5430R centrifuge (Eppendorf, Germany) and disrupted in binding buffer (20 mM HEPES buffer pH 7.4 containing 2.0 M NaCl, 10% v/v glycerol, protease inhibitor cocktail, cOmplete from Roche, Germany and 30 mM imidazole) using a sonicator (Model Q500, QSONICA, Newtown, CT, United States) with a 1.9 cm probe (Thermo Scientific, Waltham, United States). Cell debris were removed by centrifugation (25,000 × *g*, 4°C, 10 min) in an Avanti J-26 XP centrifuge (Beckman Coulter, High Wycombe, United Kingdom) and the resulting crude extract was filtered through a 0.2 μm Nalgene membrane filter (Thermo Scientific, Rockford, IL, United States). The supernatant was loaded at a flow rate of 1.0 ml/min onto a 5-ml HiTrap Ni^2+^ chelating column (GE Healthcare Life Sciences, Piscataway, NJ, United States) pre-equilibrated with binding buffer. The column was washed with binding buffer, and the protein was eluted by increasing concentration of imidazole (30–300 mM) in binding buffer. The purified active fractions were combined and further purified and concentrated with Amicon^®^ Ultra-4 Centrifugal Filter Units, 10 kDa (Cat no. UFC803024, Merck Millipore, MA, United States). Protein concentration was determined using the NanoDrop 2000c (Thermo Fisher Scientific, Pittsburgh, PA, United States) with an absorption coefficient of 9,970 M^–1^ cm^–1^ (Gasteiger et al., 2005).

### Polyacrylamide Gel Electrophoresis

The SDS-PAGE analysis was performed by using the precast Novex^®^ Tris-glycine gels (4–20%, Invitrogen, Carlsbad, CA, United States). The gels were stained with Imperial Protein Stain and Invision His-Tag In-gel stain (Thermo Fisher, Rockford, IL, United States).

### Identification of Purified Protein by LC-MS/MS Analysis

The pure samples (10 μg) were digested with trypsin/LysC mix (Promega, Madison, WI, United States) using the FASP protocol (Wiśniewski et al., 2009). Peptides were measured using a LTQ-Orbitrap mass spectrometer (Thermo Fisher Scientific, Waltham, MA, United States) and analyzed using MASCOT v2.3 (Matrix Sciences Ltd, United Kingdom).

### Activity Measurements

Carbonic anhydrase activity was measured by the SX20 Stopped-Flow Spectrometer (Applied Photophysics, Leatherhead, United Kingdom) using the pH indicator dye phenol red as described previously (Alber et al., 1999). Briefly, chamber A contained 100 μM phenol red in 20 mM MOPS buffer pH 9.8 containing 3 M KCl, with and without protein for catalyzed and uncatalyzed reaction, respectively. Chamber B contained CO_2_-saturated water prepared by bubbling CO_2_ into distilled deionized water at 25°C. The reaction was monitored spectrophotometrically by measuring the increase in absorbance at 557 nm. All slit widths were set at 0.5 mm. An attached water bath regulated the temperature as indicated. A carbonic anhydrase (0.5 μM) from bovine erythrocytes (Sigma, St. Louis, MO, United States) was used as a positive control. As a negative control, the crude lysate of untransformed *Halobacterium* sp. NRC-1Δ*ura*3Δ*icf*A cells was collected, purified using a Ni-NTA column, and the activity was measured for both the crude lysate and the purified fractions.

The specific activity was calculated via the Wilbur-Anderson unit (WAU) per 1 mg of protein, with one unit of activity being defined as (T_0_ − T)/T, where T_0_ (uncatalyzed reaction) and T (catalyzed reaction) are recorded as the time (sec.) required for the pH to drop from 9.8 to the transition point of the dye in a buffer control and in presence of enzyme or positive/negative control, respectively. Therefore, the reaction time until the activity plateau was reached was measured for the background reaction and all measurements were averaged and analyzed via the Excel solver.

### X-ray Crystallography

For crystallization, the protein was dialyzed against 50 mM Tris-HCl buffer pH 8.0, containing 300 mM NaCl and concentrated to 17 mg/ml with Amicon^®^ Ultra-4 Centrifugal Filter Units, 10 kDa (Cat no. UFC803024, Merck Millipore, MA, United States). Protein crystals were grown at 20°C using the hanging drop vapor diffusion method. The protein solution was mixed in a 1:1 ratio with the reservoir buffer, 0.1 M HEPES pH 7.5 containing 0.05 M cadmium sulfate and 0.8 M sodium acetate. Crystals selected for measurement were flash-frozen in liquid nitrogen after soaking in cryobuffer (70%, v/v reservoir buffer and 30%, v/v glycerol).

### Data Collection, Structure Solution and Refinement

Native diffraction datasets were collected at 2.6 Å resolution using synchrotron radiation at the X06SA-beamline, SLS, Villigen, Switzerland (see Supplementary Table S1). Recorded reflections were processed with XDS (Kabsch, 1993). CA_D crystallized in the cubic space group F432 with α = 362.6 Å, indicating five γ-CA-subunits in the asymmetric unit and a solvent content of 74%. Phases were obtained by Patterson search algorithms using the coordinates 1V3W as starting model (Jeyakanthan et al., 2008). The primary sequence was placed into the 2F_*o*_-F_*c*_ electron density map using COOT (Emsley and Cowtan, 2004) and refined with REFMAC5 (Murshudov et al., 1997). The model was completed in iterative rounds where temperature factors were anisotropically refined by translation/libration/screw motion-parameters, yielding crystallographic values of *R*_*cryst*_ = 0.177 and *R*_*free*_ = 0.204 (see Supplementary Table S1). Coordinates were confirmed to have adequate stereochemistry in the Ramachandran plot with 98.0% of residues in most favored, 1.8% in additionally allowed, and 0.2% in outlier regions. The crystal structure was deposited at the RCSB Protein Data Bank under accession codes 6SC4.

### Structural Analysis

Crystal structure analysis and visualization were performed using programs PyMOL (The PyMOL Molecular Graphics System, Version 1.2r3pre, Schrödinger, LLC)^1^ and Yasara (Krieger and Vriend, 2014), and SwissPDB Viewer 4.1 (Guex and Peitsch, 1997). Homolog searches were performed using the DALI server (Holm and Rosenstrom, 2010). For comparison of CA_D to homolog structures, all duplicates or structure variants belonging to the same homolog protein were neglected. Interactions between protein residues were calculated using Yasara (Krieger and Vriend, 2014), except for salt bridges which were calculated using VMD (Humphrey et al., 1996) with an oxygen-nitrogen cut-off of 4 Å (between residues Arg/Lys/His and Asp/Glu) and hydrogen bonds being estimated using Chimera (Pettersen et al., 2004).

Electrostatic surface potential calculations were performed using the PDB2PQR server (Dolinsky et al., 2004) along with the PyMOL plugin APBS (Baker et al., 2001). Surface-exposed amino acids were determined using Swiss PDB viewer 4.1 (Guex and Peitsch, 1997) (≥10% surface accessibility). An estimation of the oligomeric assembly was performed using the program PISA (Krissinel and Henrick, 2007).

### Mutation Library

The QuikChange Site-Directed Mutagenesis Kit (Agilent Technologies, Santa Clara, CA, United States) was used according to manufacturer’s instructions in combination with the primers listed in the Supplementary Table S5. pRK.CAD vector containing the CA_D gene was used as a DNA template.

## Results and Discussion

The remarkable stability of extremozymes and potential applications under harsh operational conditions has gained increased interest (Littlechild, 2017). Nonetheless, to gain a better understanding of the halophilicity in salt adapted proteins and the underlying molecular mechanisms of halophilic enzymes, additional studies of model proteins are required.

### Generation of the CA_D Protein From SAG Analysis of a Discovery Deep Sample

The CA_D gene originated from SAG analysis was identified and annotated as a γ-carbonic anhydrase (γ-CA) using the PPM algorithm (Grotzinger et al., 2014). We used the genetically modifiable extreme haloarchaeon *Halobacterium* sp. NRC-1 (Karan et al., 2013) as an expression system. *Haloarchaea* contain an internal salt concentration of 4-5 M and maintain an isoosmotic balance of ion concentrations in the cytosol with the surrounding medium. *Halobacterium* sp. NRC-1 harbors a carbonic anhydrase gene, *icf*A, located in the chromosome, location 638911 ← 639570 (Supplementary Figure S1). To eliminate background carbonic anhydrase production, *icf*A was knocked out (Berquist et al., 2006). The resulting *Halobacterium* sp. NRC-1Δ*ura*3Δ*icf*A deletion strain was used for the CA_D expression. The enzyme was purified to homogeneity, with a total yield of 5 mg of protein/liter of culture (Supplementary Figure S2A). The identity of CA_D was confirmed by tryptic digest and LC-MS/MS analysis (89% coverage, Supplementary Figure S2B).

### Crystal Structure of a Halophilic γ-Carbonic Anhydrase From the Discovery Deep Brine Pool

**FIGURE 1:**
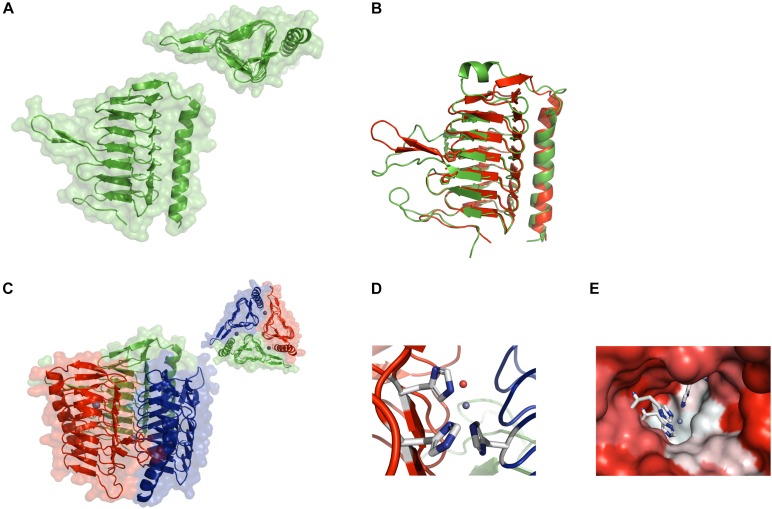
Crystal structure of CA_D. **(A)** Ribbon structure of the CA_D monomer with the background showing the water-accessible surface. The inset shows a top view of the monomer depicting the triangular shape formed by the seven-turn, left-handed β-helix. **(B)** Structural overlay of the CA_D monomer (green) and the γ-CA Cam from *Methanosarcina thermophila* (red) (PDB ID 1qrg). **(C)** Ribbon structure of the CA_D trimer along with the water-accessible surface in the background. The inset contains a top view of the trimer. The three zinc ions are depicted as gray spheres. **(D)** The CA_D active center made up of three histidine residues (white-and-blue sticks) from two adjacent monomers. The zinc ion and a water molecule are depicted as gray and red spheres, respectively. **(E)** The active site cavity is colored according to hydrophobicity (red: hydrophilic, white: hydrophobic). The coordination residues are represented as sticks.

The crystal structure of CA_D was solved successfully to a resolution of 2.6 Å (PDB ID: 6SC4). Data collection and crystallographic quality statistics are shown in Table S1. The monomer contains a seven-turn, left-handed β-helix connected to an α-helix running antiparallel to the β-helix axis (Figure 1A). The majority of the structure comprises β-sheet (54.5%), while only a minimal amount consists of α-helix (13.5%), turn (12.9%), and coil structure (19.1%). The overall structure is highly conserved compared to reported γ-Cas (Figure 1B). Whereas the protein core is common to published γ-CA structures, differences in the connection of the helix motif is conspicuous. For example, CA_D contains a connecting β-sheet, while Cam consists of an additional α-helix instead. However, the comparison of CA_D with all 10 crystallized γ-CA structures revealed that the majority contained a β-sheet with the thermophilic Cam and carboxysomal CcmM being an exception. Since this characteristic feature did not correspond to all investigated thermophilic homologs, we aimed to analyze whether the ß-sheet has a role in the stability and rigidity of the overall architecture. Furthermore, it is likewise unclear if this plays a prominent role for the enzyme function.

Notably, CA_D is organized into trimers (Figure 1C), resembling the reported active conformation for γ-CAs, where the active site includes residues from the adjacent monomers (Ferry, 2010). Closer inspection of the active site reveals a zinc ion, coordinated by three histidine residues (His64 chain A, His89 chain B, and His94 chain A). A well-defined water molecule is coordinated to the zinc ion, which acts as the nucleophile in the reaction mechanism of these enzymes (Supuran, 2016) (Figure 1D). Interestingly, one half of the active site pocket exhibits a more hydrophobic character whereas the facing side is more hydrophilic (Figure 1E) (Supuran and De Simone, 2015). Taken together, the overall structure of CA_D reveals a strong conservation to reported γ-CAs, supporting the γ-CA class gene annotation. Contrary to the sequence conservation, where the alignment of the CA_D sequence with structural homologs shows a sequence identity of less than 40% for alignments with a query coverage larger than 95%, the structure is, consequently, well conserved (Supplementary Table S2).

### Halophilic Adaptation of CA_D Compared to Other Non-halophilic γ-CAs

While the overall CA_D structure is conserved to other known γ-CAs, differences to non-halophilic γ-CAs must provide the observed stability under high salt concentrations. Thus, the structural elucidation provides interesting insights into halophilic adaptation by comparing CA_D with meso- or thermophilic γ-CAs (Supplementary Table S2).

### Comparison of Stabilizing Interactions

Compared to the average values for meso- and thermophilic CAs, the CA_D monomer contained an increased number of salt bridges (12 vs. an average of 10 and 9 for meso- and thermophilic homologs, respectively) (Supplementary Table S3), which is seen as a characteristic of haloadaptation (Frolow et al., 1996; Britton et al., 2006; Karan et al., 2012a). Despite the higher average number of salt bridges for the CA_D monomer, several homologs showed a comparable or increased amount of salt bridges. In fact, the discrepancy between the homologs within the group is large. Conversely, thermophilic homologs averaged a higher number of pi–pi interactions and an increased number of hydrogen bonds and hydrophobic interactions within the monomer that displayed an increased rigidity to maintain their structure (calculated numbers are presented in Supplementary Table S3). Oligomerization is expected to have a stabilizing effect and the calculation of the interactions within the trimers for CA_D and homologs demonstrates higher amount of interactions compared to monomers alone. Interestingly, while the CA_D monomer does not show a statistically significant increase in interactions compared to single homologs, the trimerization of CA_D stabilizes the enzyme by additional interactions on the monomer interfaces to a higher extent than for homologs. The CA_D trimer displayed an increased number of hydrogen bonds and salt bridges compared to all of the individual meso- and thermophilic homologs being additionally added to the enzyme upon trimerization (hydrogen bonds: 69 vs. homolog average of 42; salt bridges: 10 vs. a homolog average of 5 or 5.5). As the – CA_D trimer constitutes the active form, this increased stabilization is critical to assemble the active enzyme under high salt conditions.

### Comparison of the Surface-Charge

**FIGURE 2:**
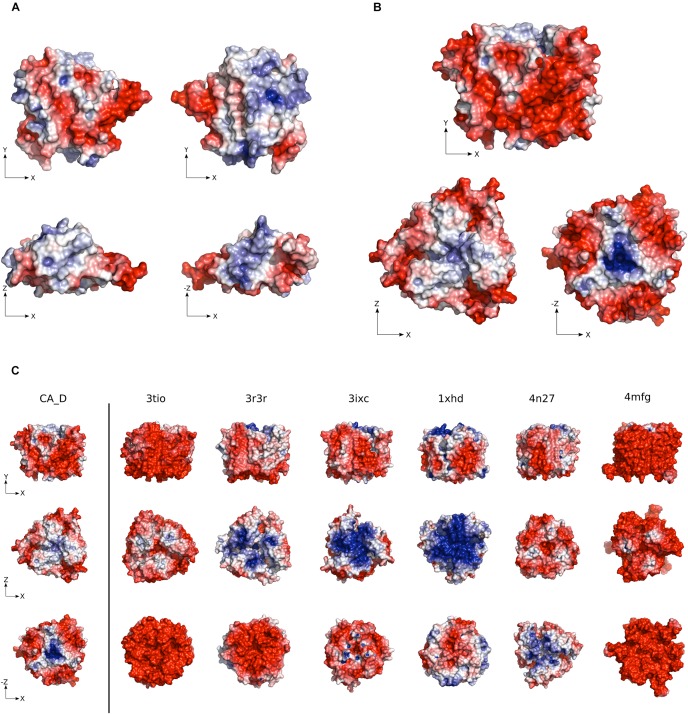
Electrostatic surface potential of CA_D. **(A)** CA_D Monomer and **(B)** CA_D Trimer electrostatic surface potential color-coded from red (negative potential) to blue (positive potential). **(C)** The surface potential of CA_D compared to mesophilic γ-CA homologs (*Escherichia coli*, 3tio; *Salmonella enterica*, 3r3r; *Anaplasma phagocytophilum*, 3ixc; *Bacillus cereus*, 1xhd; *Brucella abortus*, 4n27; *Clostridium difficile*, 4mfg). Unit: –5 to +5 k_*b*_T/e (k_*b*_ as the Boltzmann constant, T as the temperature in Kelvin and e as the charge of an electron).

Halophilic proteins are typically characterized by a highly negative charged electrostatic surface (DasSarma and DasSarma, 2015). Interestingly, while the CA_D monomer shows both positive and negative charges on its surface, the overall change is slightly more negative (pI ∼6.8). The highest negative charge is found on the surface of the flexible β10–β11 loop extending from the β-helix (Figure 2A). The presence and sequence of this loop differs between homolog γ-CAs (Park et al., 2012). For CA_D it shows a high acidic surface charge. A highly acidic surface is an important and common trait of halophilic proteins, enabling protein hydration under high salt conditions (Frolow et al., 1996; Britton et al., 2006; Grotzinger et al., 2018).

The CA_D trimer contains an overall negative surface potential, while a positive charge is located only in two concentrated locations. The positive charge is possibly involved in the fast release of the bicarbonate product. The overall negative charge indicates the burying of positive patches found on the monomer surface (Figure 2B). Compared to mesophilic homologs, CA_D showed a slightly higher negative surface charge except for *E. coli* and *C. difficile* γ-CAs (PDB ID: 3tio, 4mfg) (Figure 2C and Supplementary Figure S3). Still, this negative surface-charge is lower than often described for halophilic proteins and possibly explains the flexibility that enables stability even at comparatively low salt concentrations.

### Comparison of the Surface-Exposed Amino Acids

**FIGURE 3:**
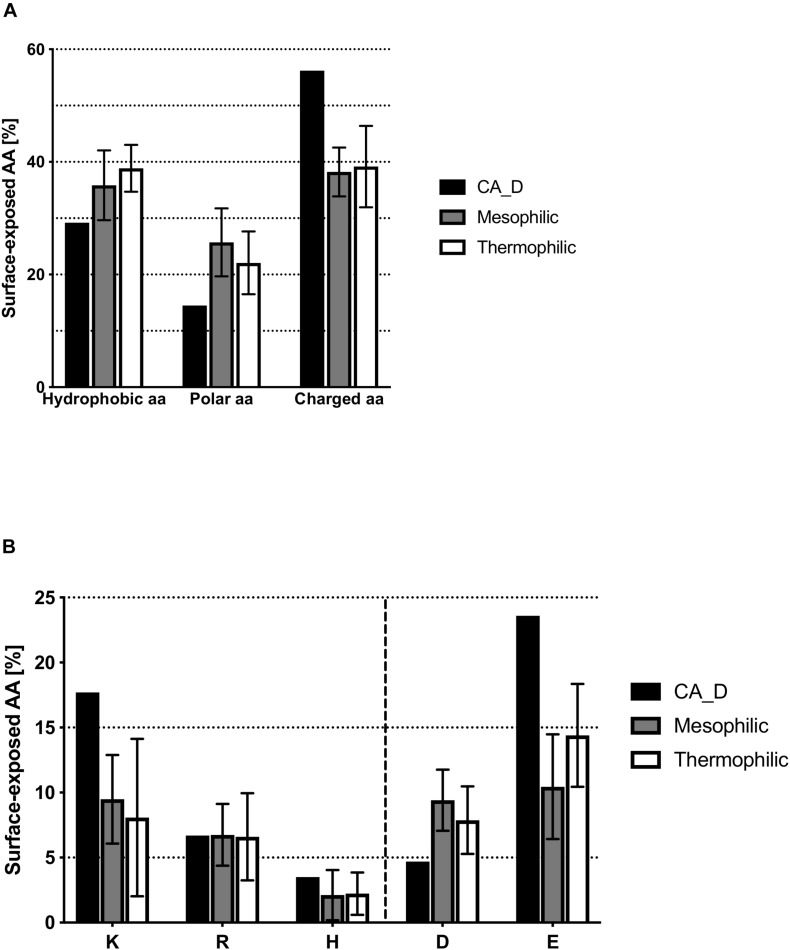
Comparison of surface-exposed amino acids between CA_D and meso- and thermophilic homologs. **(A)** hydrophobic, polar or charged amino acids residues, **(B)** depicts the amount per individual charged amino acid.

The most pronounced difference found for CA_D compared to homologs is the surface amino acid composition. The CA_D trimer protein surface contains a decreased number of hydrophobic and polar amino acid residues (Supplementary Figure S4) whilst showing an increased number of charged amino acid residues on the surface as compared to homologs. 56% of the amino acids on the CA_D protein surface are charged, compared to 38 and 39% in meso- and thermophilic homologs, respectively (Figure 3A). This decrease in hydrophobic amino acids leads to a decrease of the hydrophobic surface patch that facilitates the aggregation of the protein in CA_D (hydrophobic amino acids constitute 29% of the surface amino acids compared to an average of 36 and 39% for meso- and thermophilic homologs, respectively). An increased amount of charged amino acids is observed that form a stable hydration shell. This is essential for stability under high salt conditions. A closer look at positively and negatively charged amino acids on the trimer surface of CA_D reveals the dominance of glutamate (23.6 vs. 10.5 and 14.4% on the surface of mesophilic and thermophilic homologs, respectively) (Figure 3B). Interestingly, CA_D also contains a comparatively high amount of lysines.

The increase of surface glutamic acid residues described for CA_D is a characteristic hallmark of halophilic enzymes (DasSarma et al., 2006), as these residues have a high water-binding capacity via interaction with Na^+^ or K^+^ ions, and thereby attract the bound hydrating water (Trevino et al., 2007). This explains how the strong increase of glutamic acid on the surface of CA_D has such a pronounced effect on maintaining stability and activity at high salt concentrations. However, contradictory to reported halo-adaptation strategies, CA_D also shows increased surface lysine residues, compared to homologs. This explains why the negative surface charge is not as pronounced as for other reported halophilic proteins (Premkumar et al., 2005; Karan et al., 2012a; DasSarma et al., 2013). Lysine tends to disrupt the formation of an ordered hydration shell under elevated salt concentrations (Britton et al., 2006; Esclapez et al., 2007). At low ionic strength, the higher amount of glutamic acid on the surface causes electrostatic repulsion and, therefore, destabilizes the protein (Kohn et al., 1995; Frolow et al., 1996; Britton et al., 2006). Moreover, the long hydrophobic part of the lysine residues potentially plays a role in attracting hydrophobic CO_2_ substrate for the catalytic reaction. Noteworthy, the negatively charged glutamate residues are located around the trimer surface, except for the top and bottom view of the multimer where positive patches stem from a network of arginine residues which are possibly initiating the rapid release of the formed product (Smith et al., 2002; Tripp et al., 2002).

### CA_D Variant Library Design

**FIGURE 4:**
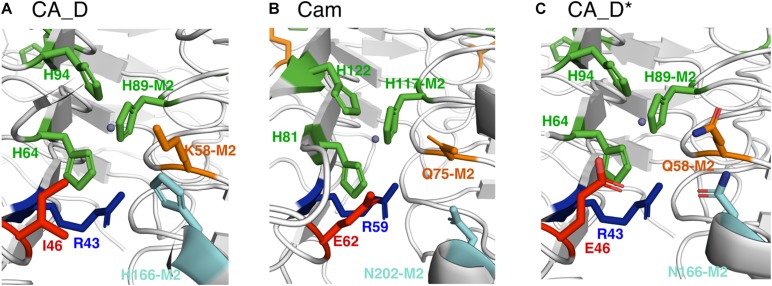
Comparison of selected active site residues. **(A)** CA_D (crystal structure), **(B)** Cam (crystal structure) from *M. thermophila* (PDB ID: 1qrg), and **(C)** CA_D* including selected mutations such as I46E, K58Q, H166N (M2: indicates that residue belongs to the adjacent monomer). The central zinc ion is depicted as a gray sphere.

Since the discovery of the first γ-CA in 1994 (Alber and Ferry, 1994), several γ-CAs have been characterized: some with a high reported activity and some with no detectable activity, raising the question whether essential residues are missing or an alternative function is appropriate (Macauley et al., 2009). We bioengineered the CA_D active center based on the CA_D crystal structure. To investigate the role of selected residues in the active site of CA_D, several variants were expressed, purified, and assayed for enzymatic activity. The selection of residues for mutagenesis was performed based on structural comparisons to γ-CA homologs as well as literature reports based on presumptions of conserved residues of γ-Cas (Smith et al., 1999; Iverson et al., 2000; Jeyakanthan et al., 2008; Ferry, 2010; Pena et al., 2010; Park et al., 2012; Ragunathan et al., 2013; Frost and McKenna, 2014). Thereby, the main comparison was focused on the Cam structure (Kisker et al., 1996). A structural comparison of the active center of CA_D (Figure 4A) with a simplified view of the Cam homolog (Figure 4B) revealed that CA_D residue I46 corresponds to Cam residue E62. This part of the enzyme plays an important role in product release, relaying protons during hydroxide formation from the zinc-bound water as well as forming hydrogen bonds with the bicarbonate. CA_D residues K58 and H166 are substituted by Q75 and N202 in Cam. These residues presumably orchestrate the orientation of the carbon dioxide for the nucleophilic attack in Cam. Moreover, Cam N202 together with E62 is thought to form hydrogen bonds with the product bicarbonate (Ferry, 2010; Pena et al., 2010). The strict conservation of the E84 position is a matter of debate, as the CamH subclass has lost this residue (Ferry, 2010) and a mutagenesis study exhibits activity for the D84 and H84 substituted Cam variants (Tripp and Ferry, 2000). The presence of D residues instead of E is explained by the different abundance of aspartic and glutamic acid in thermophilic proteins, compared to mesophilic proteins (Lee et al., 2004). Thus, the selected point mutations were I46E, K58Q, H166N, I46E-K58Q, K58Q-H166N, I46E-H166N as well as a triple CA_D^∗^ (I46E-K58Q-H166N) (Figure 4C) and a quadruple CA_D^∗^-D67E (I46E-K58Q-H166N-D67E) variant (Table 1 and Supplementary Figures S5, S6).

**TABLE 1:** Rationalization of CA_D variants.

**Constructs**	**Residues**							
1QRG (Cam)	R59	E62	Q75	E84	N202	H81	H117	H122
CA_D	R43	**I46**	**K58**	D67	**H166**	H64	H89	H94
I46E	R43	**E46**	K58	D67	H166	H64	H89	H94
K58Q	R43	I46	**Q58**	D67	H166	H64	H89	H94
H166N	R43	I46	K58	D67	**N166**	H64	H89	H94
I46E-K58Q	R43	**E46**	**Q58**	D67	H166	H64	H89	H94
K58Q-H166N	R43	I46	**Q58**	D67	**N166**	H64	H89	H94
I46E-H166N	R43	**E46**	K58	D67	**N166**	H64	H89	H94
CA_D* (I46E-K58Q-H166N)	R43	**E46**	**Q58**	D67	**N166**	H64	H89	H94
CA_D*-D67E	R43	**E46**	**Q58**	**E67**	**N166**	H64	H89	H94

*The residues in bold were modified based on the alignment with Cam shown in the first row.*

### Carbonic Anhydrase Activity Measurement of CA_D and Variants

To evaluate the potential impact of the mutations, we examined the CA_D variants for their activities expressed in Wilbur-Anderson Unit per 1 mg of protein (WAU/mg) (Wilbur and Anderson, 1948; Hou et al., 2019). The colorimetric carbonic anhydrase activity assay by Wilbur-Anderson measures the time required for a saturated CO_2_ solution to lower the pH of a specific buffer. Respective controls were: (i) a carbonic anhydrase from bovine erythrocytes as a positive control; and (ii) the crude lysate of untransformed *Halobacterium* sp. NRC-1Δ*ura*3Δ*icf*A cells as negative controls (Supplementary Figure S7). Purified lysate and the crude lysate did not show any enzyme activity. Therefore, these measurements confirmed that the observed activity resulted from the purified CA_D variants. As CA_D is from an uncultured archaeon from the Discovery Deep brine pool, the enzyme activity was measured at 40°C in a solution containing 3 M KCl.

**FIGURE 5:**
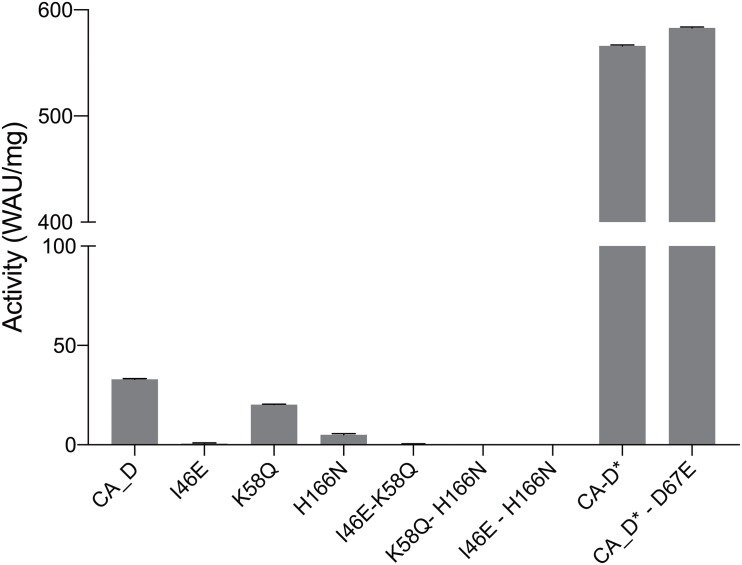
Carbonic anhydrase activity assessment. CA_D WT and variants measured at 3 M KCl and 40°C.

The activity assays displayed a distinct profile for CA_D with a rather low activity of ∼33 WAU/mg. In contrast, the engineered variants showed a decreased activity for I46E, K58Q, and H166N, and undetectable activity for the double variants, I46E-K58Q, K58Q-H166N, and I46E-H166N (Figure 5 and Supplementary Table S4). However, the triple variant CA_D^∗^ (I46E-K58Q-H166N) displayed a 17-fold increased activity of 566 WAU/mg (Figure 5 and Supplementary Table S4) compared to CA_D.

These results are in close alignment with previous findings of related γ-carbonic anhydrases (γ-CAs). In the most prominent γ-CA Cam, residue E84 has been proposed as part of a proton shuttling network along with E62, E88, and E89 on the acidic loop (Tripp and Ferry, 2000). Conversely, when fully mimicking the expected proton shuttling network, activity of the quadruple variant CA_D^∗^-D67E (Figure 5) is slightly higher as for the triple variant CA_D^∗^, which is in agreement with the observations made in γ-CA Cam (Tripp and Ferry, 2000; Ferry, 2010).

Our findings support the proposed mechanism for Cam, in which the probed amino acids play a decisive role. Some conclusions, as to why CA_D is lacking activity could be drawn by comparing the altered residues in CA_D^∗^ to γ-CA Cam (Ferry, 2010). With the CA_D mutation I46E, a hydrophobic residue was replaced with a negatively charged one, the corresponding residue in the Cam homolog presumably is essential in water activation to enable the reaction (Ferry, 2010). K58Q and H166N are further substitutions based on Cam active residues and facilitate the orientation of the carbon dioxide, while the H166N substitution facilitates hydrogen bonding and release of the bicarbonate (Ferry, 2010). We, therefore, assume that CA_D is regulated by a similar proton shuttling network such as Cam (Ferry, 2010), but due to its halophilic nature CA_D behaves slightly differently.

## Conclusion

High cytoplasmic salt concentrations critically affect the folding and activity of proteins and other macromolecules as they may induce protein aggregation due to enhanced hydrophobic interactions, increased hydration of ions, decreased availability of unbound water molecules, and prevention of intra- and intermolecular electrostatic interactions (Karan et al., 2012a, b). Halophilic proteins are adapted to maintain their native conformation under high salt concentrations. They are functionally active in the presence of high salt concentrations, following halo-adaptation strategies such as high acidic amino acid content on the surface, low hydrophobicity at the core of the protein, and an increased number of salt bridges (Madern et al., 2000; Fukuchi et al., 2003; Bolhuis et al., 2008; Tadeo et al., 2009; DasSarma et al., 2013).

We used single amplified genomes to resuscitate a γ-carbonic anhydrase (γ-CA) from an uncharacterized haloarchaeon collected from a brine pool at the bottom of the Red Sea. The detailed structural analysis and comparison with previously solved structures of mesophilic γ-CAs revealed the molecular features of its extremophilic nature, caused by the unique habitat. The most prominent features of extremophilicity are the increased charged residues on the protein surface and an increased number of hydrogen bonds as well as salt bridges. Investigation of CA_D, therefore, demonstrates potential for further development and implementation of SAG analysis to generate extremozymes from previously inaccessible environments. This approach has to date only been scarcely used to characterize specific proteins of interest (Grotzinger et al., 2018). Further, mutation analysis provided an interesting insight into active site residue conservation for γ-CAs and enabled us to increase the CA_D function by 17-fold. Moreover, the applied combination of mechanistic insights from the thermophilic Cam into the scaffold of the halophilic CA_D, resulting in the CA_D^∗^ variant which demonstrated high activity and stability, underlines the potential of protein evolution for extremophilic proteins for industrial applications and the design of novel catalysts for industry.

## Data Availability Statement

The datasets generated for this study can be found in the PDB ID: 6SC4.

## Author Contributions

MR and JE conceived and supervised the study. MV and RK designed and performed the experiments with the help of DR and AV. SG, PD, and SD provided plasmid and support. M-TV and MG solved the crystal structure. MV and RK wrote the manuscript. All the authors contributed to and commented on this manuscript.

## Conflict of Interest

The authors declare that the research was conducted in the absence of any commercial or financial relationships that could be construed as a potential conflict of interest.

## Footnotes

1 https://bioinformaticschap.wordpress.com/tools/visulization-tools/pymol/
