# Supplementary material for: Crystal Structure and Active Site Engineering of a Halophilic γ-Carbonic Anhydrase
Source: Front Microbiol. 2020 Apr 28;11:742. doi: 10.3389/fmicb.2020.00742 (PMC7199487; doi:10.3389/fmicb.2020.00742)
Supplement: Supplementary file 1 [file Data_Sheet_1.pdf]

Crystal structure and active site engineering of a halophilic γ-carbonic anhydrase

Malvina Vogler[1,2,∞], Ram Karan[1,*,∞], Dominik Renn[1], Alexandra Vancea[1], Marie-Theres Vielberg[2], Stefan W. Grötzinger[1], Priya DasSarma[3], Shiladitya DasSarma[3], Jörg Eppinger[1], Michael Groll[2*], Magnus Rueping[1*]

[1]King Abdullah University of Science and Technology (KAUST), KAUST Catalysis Center (KCC), Thuwal 23955-6900, Saudi Arabia

[2]Technische Universität München (TUM), Center for Integrated Protein Science Munich at the Department Chemistry, Garching 85748, Germany

[3]Department of Microbiology and Immunology, University of Maryland School of Medicine, and Institute of Marine and Environmental Technology, Baltimore, MD 21202 USA

Table S1. Crystallographic data collection and refinement statistics.

	γ-CA
**Crystal parameters**	
Space group	F432
Cell constants	a= 362.4 Å
Subunits / AU[a]	5
**Data collection**	
Beam line	X06SA, SLS
Wavelength (Å)	1.28
Resolution range (Å)[b]	50 - 2.6 (2.7 - 2.6)
No. observed reflections	848,244
No. unique reflections[c]	118535
Completeness (%)[b]	100 (100)
R_{merge} (%)[b, d]	8.5 (57.5)
I/σ (I)[b]	18.8 (3.3)
**Refinement (REFMAC5)**	
Resolution range (Å)	30 - 2.6
No. refl. working set	59,365
No. refl. test set	3,124
No. non hydrogen	7,548
No. of metals	25
Solvent	611
R_{work} / R_{free} (%)[e]	17.7 / 20.4
r.m.s.d. bond (Å) / (°)[f]	0.006 / 1.25
Average B-factor (Å^2)	47.5
Ramachandran Plot (%)[g]	98.0 / 1.8 / 0.2
PDB accession code	6SC4

[a] Asymmetric unit

[b] The values in parentheses for resolution range, completeness, R_{merge} and I/σ (I) correspond to the highest resolution shell

[c] Data reduction was carried out with XDS and from a single crystal. Friedel pairs were treated as individual reflections

[d] $R_{merge}(I) = \Sigma_{hkl}\Sigma_j \mid I(hkl)_j - \langle I(hkl)\rangle \mid / \Sigma_{hkl} \Sigma_j I(hkl)_j$, where $I(hkl)_j$ is the jth measurement of the intensity of reflection hkl and $\langle I(hkl)\rangle$ is the average intensity

[e] $R = \Sigma_{hkl} \mid |F_{obs}| - |F_{calc}| \mid / \Sigma_{hkl} |F_{obs}|$, where R_{free} is calculated without a sigma cut off for a randomly chosen 5% of reflections, which were not used for structure refinement, and R_{work} is calculated for the remaining reflections

[f] Deviations from ideal bond lengths/angles

[g] Percentage of residues in favored region / allowed region / outlier region

Table S2. Homolog γ-CA structures as found and ranked by the DALI Server (Holm and Rosenstrom, 2010). The proteins are classified according to their host organism being meso- or thermophilic and their sequence identity to CA_D is given. (Note: homolog CA with the PDB ID 3r1w is not included as its source is unknown but presumably stems from a psychrophilic organism. As this is the only example for this class it is not included for statistical reasons.)

PDB ID	RMSD(Holm and Rosenstrom, 2010)	Organism	Type (DSMZ)	Sequence identity (Query cover) (Altschul et al., 1990; Altschul et al., 1997) [%]
3tio	0.9	*Escherichia coli*	mesophilic	27 (96)
2fko	0.8	*Pyrococcus horikoshii*	thermophilic	43 (94)
3ixc	0.7	*Anaplasma phagocytophilum*	mesophilic	36 (93)
3vnp	0.9	*Geobacillus kaustophilus*	thermophilic	32 (96)
1xhd	1.0	*Bacillus cereus*	mesophilic	29 (96)
3r3r	1.4	*Salmonella enterica*	mesophilic	28 (95)
4n27	1.0	*Brucella abortus*	mesophilic	36 (98)
4mfg	1.2	*Clostridium difficile*	mesophilic	37 (95)
1qrg	2.6	*Methanosarcina thermophila*	thermophilic	31 (75)
3kwc	1.5	*Thermosynechococcus elongatus*	thermophilic	43 (82)

Table S3. Interactions of CA_D and homolog γ-CA structures. The number of interactions is indicated within each monomer on the left side and the ones that are added by trimerization, meaning the interactions which were additionally added upon trimer formation, are displayed on the right side (HB: Hydrogen Bonds, SB: Salt Bridges). Hereby, CA_D is compared to the average number of interactions in mesophilic and thermophilic homologs, while the single numbers per homolog are listed below each average. The highest value per row (CA_D compared to the average values) is marked as **bold**. In the case of a substantial difference between CA_D, the average values or the individual homologue values, the value is marked as **<u>bold and underlined</u>**.

Enzyme	Contacts within monomer					Influence of trimerization				
	HB	SB	pi-pi	cation-pi	Hydro-phobic	HB	SB	pi-pi	cation-pi	Hydro-phobic
**CA_D**	162	**12**	34	**18**	1014	**<u>69</u>**	**<u>10</u>**	60	33	350
**Mesophilic average**	167	10	35	12	963	42	5,5	**68**	33	324
3tio	162	13	39	16	1058	26	7	103	40	265
3ixc	173	8	39	11	892	36	3	45	30	294
1xhd	190	12	34	8	974	49	6	67	30	418
3r3r	183	15	24	20	1009	58	9	81	24	323
4n27	145	6	38	12	897	49	5	68	46	399
4mfg	149	8	33	6	945	31	3	46	29	243
**Thermophilic average**	**172**	9	**52**	15	**1036**	42	5	58	**34**	**392**
2fko	153	9	58	16	1068	45	6	27	72	342
3vnp	159	14	28	8	944	44	3	62	53	365
1qrg	193	6	52	12	1109	54	6	30	6	381
3kwc	183	6	69	24	1024	23	6	111	3	481

Table S4. Activity of CA_D variants.

	WT	Single mutants			Double mutants			Triple mutant	Quadruple Mutant
	CA_D	I46E	K58Q	H166N	I46E-K58Q	K58Q-H166N	I46E-H166N	CA_D*	CA_D*-D67E
WAU/mg	32.93	0.65	20.21	5.09	-	-	-	566	583

Table S5. Oligonucleotides used in this study.

Primer	5′-3′sequence	Use
5'-O	CGCAAGCTTGACGTCGTGCTCTCCAGTTTC	Deletion of *icf*A gene
5'-I	GATCCGTGACGCGATTTCGGGTCTAGACTGGTTGCCCGCCAGCAT	
3'-I	CCCGAAATCGCGTCACGGATCACTCGGCTCTGGAGTTACTAA	
3'-O	CGCGAATTCACTCTCAGATCCGACGTCACG	
pRK.F	GCTGGACTGCCTTTTCTTCG	Sequencing of CA_D gene
pRK.R	GTTACTCCACCGTCATTCAGC	
RK.CAA_ I46E.1	CGATGGTGATCTTCTCCTCGTCGGCGCGGATGACG	Mutation I46E
RK.CAA_ I46E.2	CGTCATCCGCGCCGACGAGGAGAAGATCACCATCG	
RK.CAA K58Q.1	ATGACGGCGTTGTCCTGGATGCAGGTGTTCTTG	Mutation K58Q
RK.CAA K58Q.2	CAAGAACACCTGCATCCAGGACAACGCCGTCAT	
RK.CAA H166N.1	CGAGCTCGGCGTTGGTGTCGGCCTG	Mutation H166N
RK.CAA H166N.2	CAGGCCGACACCAACGCCGAGCTCG	

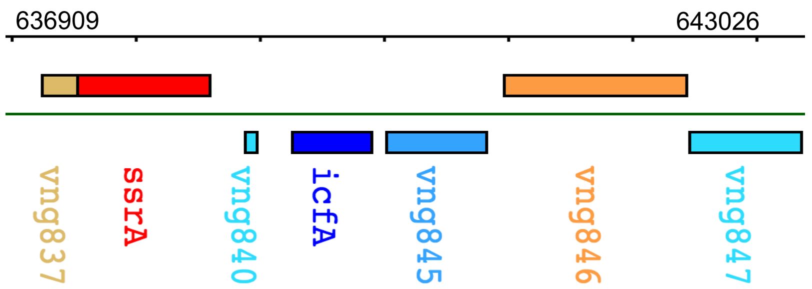

Figure S1. Location of the carbonic anhydrase gene (*icf*A) on the *Halobacterium* sp. NRC-1 chromosome (DasSarma et al., 2010).

(a)

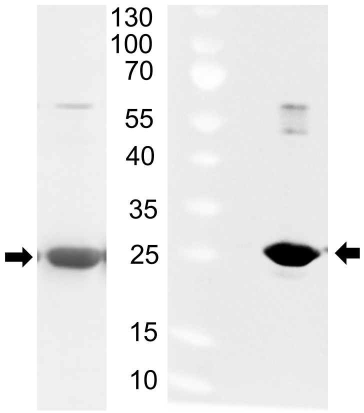

(b)

MHHHHHHMRMIQ**KFEGKKPEIHETAFVHPRATIIGDVEIGP**
KTSVWPGAVIRADIEK**ITIGKNTCIKDNAVIHPADVYHEEEIE**
YVPVKIGDNNIIGHRALIHGAK**INDESIVGAGSIVFNKAEVKT**
NSMVGMGAVVLEKQEVPNGKIVVGIPARVLRELEEREIKQIK
KQADTHAELAEHYSREIEEP

Figure S2. Purification of CA_D. (a) Purification of CA_D from *Halobacterium* sp. NRC-1Δ*ura*3Δ*icf*A by Ni-Sepharose column. Purified fractions were electrophoresed on a 4-20 % polyacrylamide–SDS gel, and stained with Coomassie blue (left) and InVision His-tag In-gel Stain (right). SDS-PAGE and InVision His-tag In-gel stain analysis revealed a prominent single band with a molecular mass of ~26 kDa (calculated molecular weight of 21.1 kDa) (Figure S2A). The high apparent molecular mass anomaly was expected based on previously reported highly acidic haloarchaeal proteins(Karan et al., 2013; Karan et al., 2014). (b) Tryptic digest and LC-MS/MS analysis of CA_D. Matched peptides are shown in *bold.* Sequence coverage was 89 %.

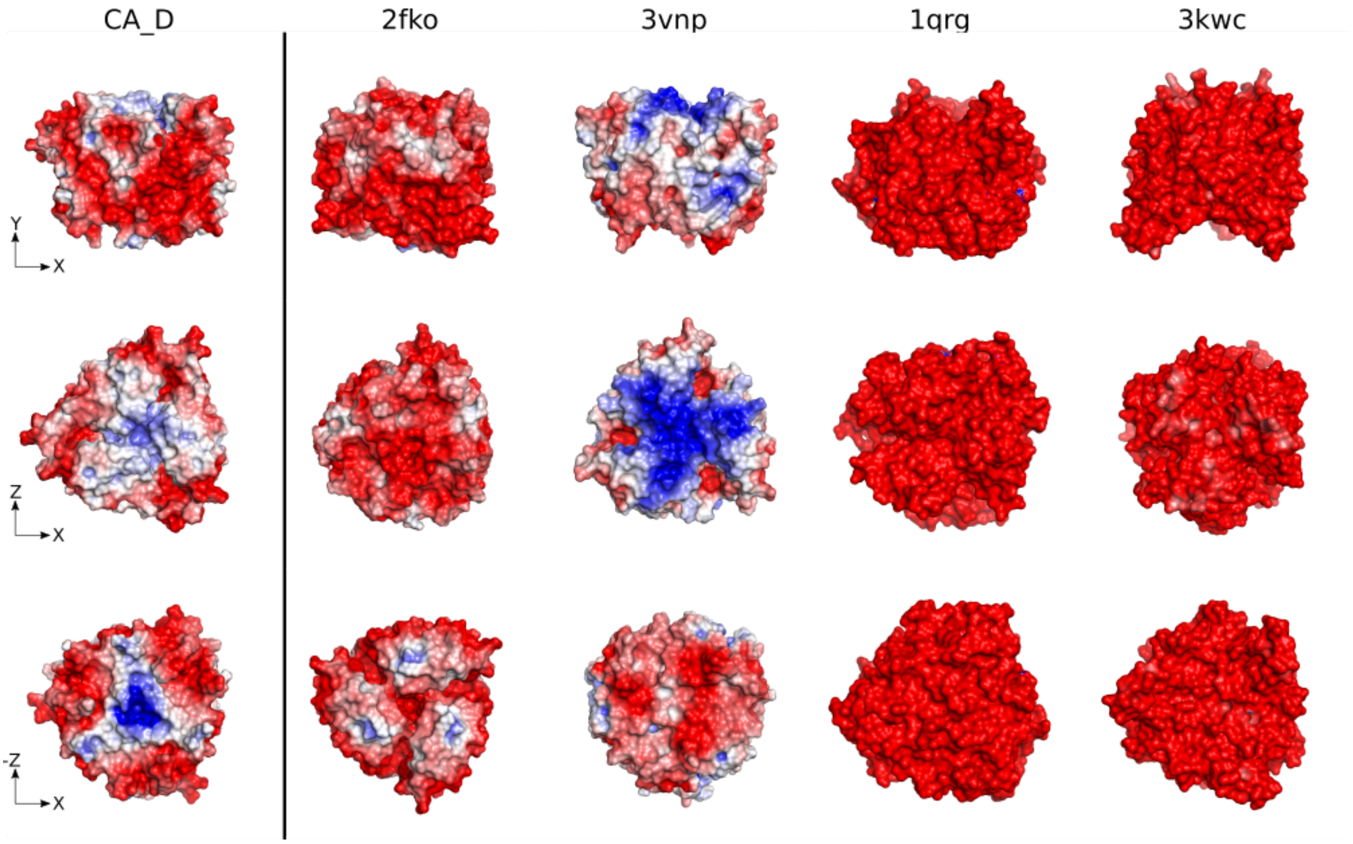

Figure S3. Comparison of the electrostatic surface potential of CA_D. The electrostatic surface potential of the CA_D trimer is compared to thermophilic homologs (*Pyrococcus horikoshii,* 2fko; *Geobacillus kaustophilus,* 3vnp; *Methanosarcina thermophile,* 1qrg; *Thermosynechococcus elongates,* 3kwc). All, except 3vnp show an increased negative surface charge. The electrostatic surface potential is color-coded from red (negative potential) to blue (positive potential). Unit -5 to +5 k_BT/e.

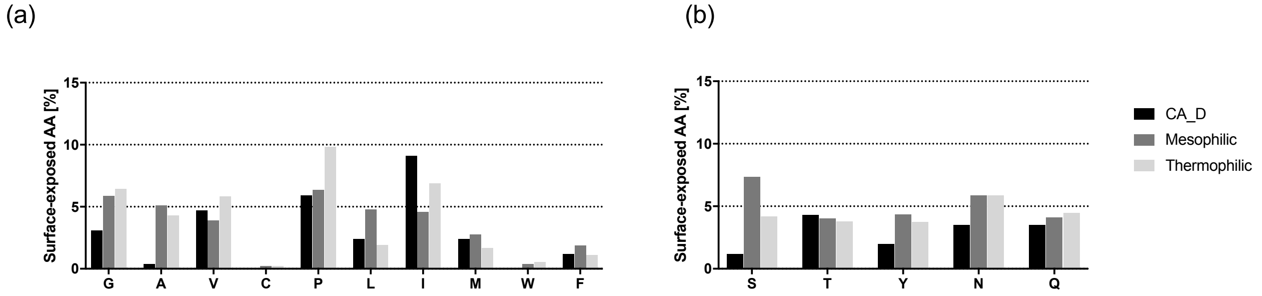

Figure S4. Comparison of amino acid composition. Single (a) hydrophobic and (b) polar surface-exposed amino acids between CA_D and meso- and thermophilic homologs.

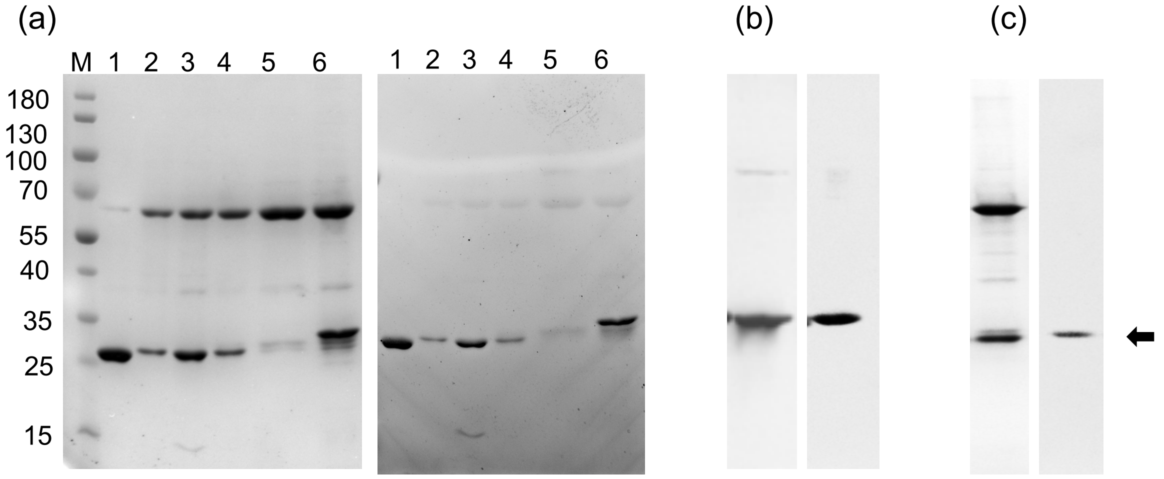

Figure S5. Purification of CA_D mutants. Purification of CA_D mutants from *Halobacterium* sp. NRC-1Δ*ura*3Δ*icf*A by Ni-Sepharose column. Purified fractions were electrophoresed on a 4-20 % polyacrylamide–SDS gel, and stained with Coomassie blue (left) and InVision His-tag In-gel Stain (right). (a) Single and double variants, Lane 1. I46E, Lane 2. K58Q, Lane 3. H166N, Lane 4. I46E-K58Q, Lane 5. K58Q-H166N, Lane 6. I46E-H166N, (b) Triple variants CA_D* (I46E-K58Q-H166N). (c) Quadrepole variants CA_D*-D67E.

I46E

MHHHHHHMRMIQK**FEGKKPEIHETAFVHPRATIIGDVEIGPKTSVWPGAVIRADEEK**
ITIGK**NTCIKDNAVIHPADVYHEEEIEYVPVKIGDNNIIGHR**ALIHGAK**INDESIVGAGSI**
VFNKAEVK**TNSMVGMGAVVLEKQEVPNGKIVVGIPARVLRELEEREIK**QIK**KQADT**
HAELAEHYSREIEEP

K58Q

MHHHHHHMRMIQK**FEGKKPEIHETAFVHPRATIIGDVEIGPKTSVWPGAVIRADIEK**
ITIGKNTCIQDNAVIHPADVYHEEEIEYVPVKIGDNNIIGHRALIHGAK**INDESIVGAGSI**
VFNKAEVKTNSMVGMGAVVLEKQEVPNGKIVVGIPARVLRELEEREIKQIK**KQADTH**
AELAEHYSREIEEP

H166N

MHHHHHHMRMIQK**FEGKKPEIHETAFVHPRATIIGDVEIGPKTSVWPGAVIRADIEK**
ITIGKNTCIKDNAVIHPADVYHEEEIEYVPVKIGDNNIIGHRALIHGAK**INDESIVGAGSI**
VFNKAEVKTNSMVGMGAVVLEKQEVPNGKIVVGIPARVLRELEEREIKQIK**KQADT**
NAELAEHYSREIEEP

I46E-K58Q

MHHHHHHMRMIQK**FEGKKPEIHETAFVHPRATIIGDVEIGPKTSVWPGAVIRADEEK**
ITIGK**NTCIQDNAVIHPADVYHEEEIEYVPVKIGDNNIIGHR**ALIHGAK**INDESIVGAGSI**
VFNKAEVKTNSMVGMGAVVLEKQEVPNGKIVVGIPARVLRELEEREIKQIK**KQADTH**
AELAEHYSREIEEP

I46E-H166N

MHHHHHHMRMIQK**FEGKKPEIHETAFVHPRATIIGDVEIGPKTSVWPGAVIRADEEK**
ITIGKNTCIKDNAVIHPADVYHEEEIEYVPVKIGDNNIIGHRALIHGAK**INDESIVGAGSI**
VFNKAEVKTNSMVGMGAVVLEKQEVPNGKIVVGIPARVLRELEEREIKQIK**KQADT**
NAELAEHYSREIEEP

K58Q-H166N

MHHHHHHMRMIQK**FEGKKPEIHETAFVHPRATIIGDVEIGPKTSVWPGAVIRADIEK**
ITIGKNTCIQDNAVIHPADVYHEEEIEYVPVKIGDNNIIGHRALIHGAK**INDESIVGAGSI**
VFNKAEVKTNSMVGMGAVVLEKQEVPNGKIVVGIPARVLRELEEREIKQIK**KQADT**
NAELAEHYSREIEEP

CA_D*(I46E-K58Q-H166N)

MHHHHHHMRMIQK**FEGKKPEIHETAFVHPRATIIGDVEIGPKTSVWPGAVIRADEEK**
ITIGKNTCIQDNAVIHPADVYHEEEIEYVPVKIGDNNIIGHRALIHGAK**INDESIVGAGSI**
VFNKAEVKTNSMVGMGAVVLEKQEVPNGKIVVGIPARVLRELEEREIKQIK**KQADT**
NAELAEHYSREIEEP

CA_D*-D67E

MHHHHHHMRMIQK**FEGKKPEIHETAFVHPRATIIGDVEIGPKTSVWPGAVIRADEEKITI**
GKNTCIQDNAVIHPADVYHEEEIEYVPVKIGDNNIIGHRALIHGAK**INDESIVGAGSIVFNK**

AEVKTNSMVGMGAVVLEKQEVPNGKIVVGIPARVLRELEEREIKQIK**KQADTNAELAEH YSREIEEP**

Figure S6. Tryptic digest and LC-MS/MS analysis of CA_D WT and variants. Mutated amino acids are highlighted in green. Matched peptides are shown in *bold.* Sequence coverage was 85-94 %.

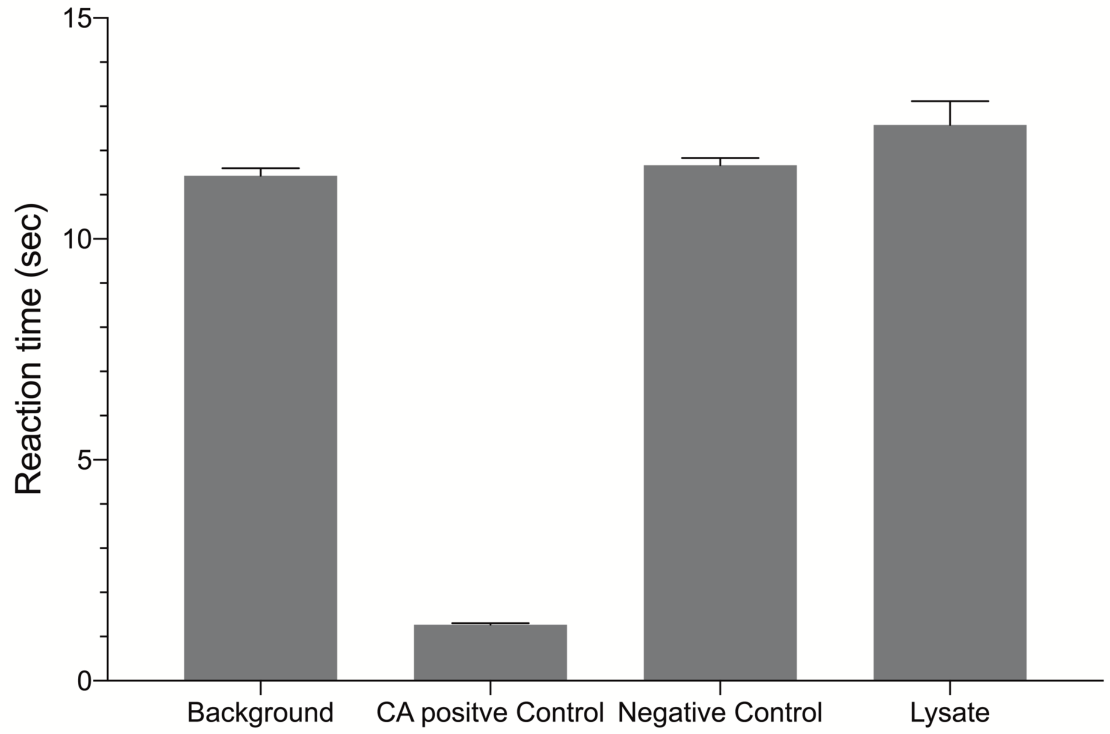

Figure S7. Control Activity Measurements. The reaction time until the activity plateau was reached was measured for the background reaction (Background) and the crude lysate (Lysate) of untransformed *Halobacterium* cells as well as for crude lysate which was purified on a Ni-NTA column (Negative) as a negative control. As a comparison, the reaction time was measured with the commercial carbonic anhydrase from bovine erythrocytes Sigma (St. Louis, MO, USA) as a positive control.

SUPPLEMENTARY REFERENCES

Altschul, S.F., Gish, W., Miller, W., Myers, E.W., and Lipman, D.J. (1990). Basic local alignment search tool. *Journal of Molecular Biology* 215(3)**,** 403-410. doi: https://doi.org/10.1016/S0022-2836(05)80360-2.

Altschul, S.F., Madden, T.L., Schäffer, A.A., Zhang, J., Zhang, Z., Miller, W., et al. (1997). Gapped BLAST and PSI-BLAST: a new generation of protein database search programs. *Nucleic Acids Research* 25(17)**,** 3389-3402. doi: 10.1093/nar/25.17.3389.

DasSarma, S.L., Capes, M.D., DasSarma, P., and DasSarma, S. (2010). HaloWeb: the haloarchaeal genomes database. *Saline systems* 6**,** 12-12. doi: 10.1186/1746-1448-6-12.

Holm, L., and Rosenstrom, P. (2010). Dali server: conservation mapping in 3D. *Nucleic Acids Res* 38(Web Server issue)**,** W545-549. doi: 10.1093/nar/gkq366.

Karan, R., Capes, M.D., DasSarma, P., and DasSarma, S. (2013). Cloning, overexpression, purification, and characterization of a polyextremophilic beta-galactosidase from the Antarctic haloarchaeon Halorubrum lacusprofundi. *BMC Biotechnol* 13**,** 3. doi: 10.1186/1472-6750-13-3.

Karan, R., DasSarma, P., Balcer-Kubiczek, E., Weng, R.R., Liao, C.C., Goodlett, D.R., et al. (2014). Bioengineering radioresistance by overproduction of RPA, a mammalian-type single-stranded DNA-binding protein, in a halophilic archaeon. *Appl Microbiol Biotechnol* 98(4)**,** 1737-1747. doi: 10.1007/s00253-013-5368-x.
